# LMWHs dosage and outcomes in acute pulmonary embolism with renal insufficiency, an analysis from a large real-world study

**DOI:** 10.1186/s12959-022-00385-z

**Published:** 2022-05-05

**Authors:** Dingyi Wang, Guohui Fan, Jieping Lei, Yuanhua Yang, Xiaomao Xu, Yingqun Ji, Qun Yi, Hong Chen, Xiaoyun Hu, Zhihong Liu, Yimin Mao, Jie Zhang, Juhong Shi, Zhu Zhang, Sinan Wu, Xincao Tao, Wanmu Xie, Jun Wan, Yunxia Zhang, Shuai Zhang, Kaiyuan Zhen, Zhonghe Zhang, Baomin Fang, Chen Wang, Zhenguo Zhai, On behalf of the China pUlmonary thromboembolism REgistry Study (CURES) investigators

**Affiliations:** 1 Institute of Clinical Medical Sciences, National Center for Respiratory Medicine, China-Japan Friendship Hospital, Beijing, China; 2 Institute of Respiratory Medicine, Chinese Academy of Medical Sciences, Beijing, China; 3 Department of Pulmonary and Critical Care Medicine, Beijing Chao-Yang Hospital, Capital Medical University, Beijing, China; 4 Department of Pulmonary and Critical Care Medicine, Beijing Hospital, National Center of Gerontology, Beijing, China; 5 Institute of Geriatric Medicine, Chinese Academy of Medical Sciences, Beijing, China; 6 Department of Pulmonary and Critical Care Medicine, East Hospital, Tongji University School of Medicine, Shanghai, China; 7 Department of Pulmonary and Critical Care Medicine, Sichuan University West China Hospital, Chengdu, China; 8 Department of Pulmonary and Critical Care Medicine, Chongqing Medical University First Affiliated Hospital, Chongqing, China; 9 First Hospital of Shanxi Medical University, First Hospital of Shanxi Medical University, Taiyuan, China; 10 Department of Cardiology, National Center for Cardiovascular Diseases, Fuwai Hospital, Chinese Academy of Medical Sciences and Peking Union Medical College, Beijing, China; 11 Department of Pulmonary and Critical Care Medicine, The First Affiliated Hospital of Henan University of Science and Technology, Luoyang, China; 12 Department of Pulmonary Critical Care Medicine, Jilin University Second Hospital, Changchun, China; 13 Department of Pulmonary and Critical Care Medicine, Peking Union Medical College Hospital, Beijing, China; 14 Department of Pulmonary and Critical Care Medicine, Center of Respiratory Medicine, China-Japan Friendship Hospital, Beijing, China; 15 National Center for Respiratory Medicine, Beijing, China; 16 Department of Pulmonary and Critical Care Medicine, Beijing Anzhen Hospital, Capital Medical University, Beijing, China; 17 Department of Pulmonary and Critical Care Medicine, First Affiliated Hospital of Dalian Medical University, Dalian, China; 18 National Clinical Research Center for Respiratory Diseases, Beijing, China; 19 Chinese Academy of Medical Sciences, Peking Union Medical College, Beijing, China; 20 Department of Respiratory Medicine, Capital Medical University, Beijing, China

**Keywords:** Acute pulmonary embolism, Renal insufficiency, Low molecular weight heparin, Adjusted dosage, Prognoses

## Abstract

**Background:**

Renal function is associated with prognoses for acute pulmonary embolism (PE).

**Objective:**

To investigate the application of anticoagulants and dosage of LMWH among patients with renal insufficiency (RI), and the association between LWMH dosage and the patients’ in-hospital outcomes.

**Methods:**

Adult patients diagnosed with non-high risk acute PE from 2009 to 2015, with available data of creatinine clearance (CCr) were enrolled from a multicenter registry in China. Renal insufficiency (RI) was defined as CCr < 60 ml/min. LMWH dosage was converted into IU/kg daily dose and presented as adjusted dose (≤ 100 IU/kg/day) and conventional dose (> 100 IU/kg/day). All-cause death, PE-related death and bleeding events during hospitalization were analyzed as endpoints.

**Results:**

Among the enrolled 5870 patients, RI occurred in 1311 (22.3%). 30 ≤ CCr < 60 ml/min was associated with higher rate of bleeding events and CCr < 30 ml/min was associated with all-cause death, PE-related death and major bleeding. Adjusted-dose LMWH was applied in 26.1% of patients with 30 ≤ CCr < 60 ml/min and in 26.2% of CCr < 30 ml/min patients. Among patients with RI, in-hospital bleeding occurred more frequently in those who were administered conventional dose of LMWH, compared with adjusted dose (9.2% vs 5.0%, *p* = 0.047). Adjusted dose of LMWH presented as protective factor for in-hospital bleeding (OR 0.62, 95%CI 0.27–1.00, *p* = 0.0496) and the risk of bleeding increased as length of hospital stay prolonged (OR 1.03, 95%CI 1.01–1.06, *p* = 0.0014).

**Conclusions:**

The proportion of adjusted usage of LMWH was low. The application of adjusted-dose LMWH was associated with lower risk of in-hospital bleeding for RI patients, in real-world setting of PE treatment. Anticoagulation strategy for RI patients should be paid more attention and requires evidence of high quality.

**Trial Registration:**

The CURES was registered in ClinicalTrias.gov, identifier number: NCT02943343.

**Supplementary Information:**

The online version contains supplementary material available at 10.1186/s12959-022-00385-z.

## Introduction

Renal insufficiency is one of the generally accepted indications of an increased mortality in various cardiovascular diseases. It has been identified as an independent risk factor for short-term and long-term all-cause mortality and other adverse outcomes in pulmonary embolism (PE) patients in recent years [1, 2]. For example, in the International Cooperative Pulmonary Embolism Registry (ICOPER) study, renal dysfunction (defined as creatinine level > 2.0 mg/dL) was predictive for mortality; in Registro Informatizado de Enfermedad TromboEmbólica (RIETE) study, renal dysfunction (defined as creatinine clearance (CCr) < 30 mL/min) was found to be independently associated with an increased risk for fatal PE and fatal bleeding within 15 days of diagnosis.

Anticoagulation therapy is the core treatment strategy for intermediate and low risk PE patients. Low molecular weight heparin (LMWH) is excreted via the kidney, indicating a potential for accumulation in patients with impaired renal function [3]. Therefore, the guidelines recommend a dose reduction or 50% in patients with moderate renal insufficiency (CCr 30–50 ml/min) [4, 5], but this recommendation was no longer mentioned in recent guidelines. Unfractionated heparin (UFH) was recommended for patients with severe renal insufficiency and an adjusted dosing scheme of LMWH should be used if prescribed in those patients [6]. Non-adherence to the guideline may lead to worse outcomes, but it has been seldom reported under real-world setting. As demonstrated by RIETE study, as high as 20.8% were non-adherent cases for severe renal insufficiency, severe obesity and unstable PE patients, which was related to high risk of death [7]. However, the prognosis of PE patients with renal insufficiency who are undertaken adjusted or unadjusted (conventional) LMWH dosages has not been studied.

In the present analysis from the China Pulmonary Thromboembolism Registry Study (CURES) (ClinicalTrias.gov identifier: NCT02943343), we aimed to investigate the application of anticoagulants and dosage of LMWH among patients with renal insufficiency (RI), and the association between LWMH dosage and the patients’ in-hospital outcomes.

## Materials and methods

### Patients and study design

The CURES is an ongoing prospective, multicenter registry of consecutive patients presenting with subjectively confirmed PE with/without deep vein thrombosis (DVT). The study design has been previously reported [8]. PE was confirmed by helical computed tomographic pulmonary angiography (CTPA), ventilation-perfusion lung scintigraphy (V/Q scan) or pulmonary angiography. Patients identified high-risk PE (shock or systemic systolic blood pressure levels < 90 mmHg), CCr unable to be calculated on admission and undertook thrombolysis therapy as initial treatment were excluded.

Decisions on the treatment pattern such as to initiate, maintain, or change treatment were at the discretion of the physicians and patients. Patients’ data were collected using the electronic data capture system. Diagnostic methods and treatment of PE were at the discretion of attending physicians of the participating centers.

Demographic data, medical history related to venous thromboembolism (VTE), risk factors for VTE, symptoms and signs on presentation, physical and laboratory examination results, image test results, types of diagnostic methods, diagnostic results, therapeutic management and clinical outcomes of PE during hospitalization were collected.

The study was approved by institutional review boards and ethical committees of all the centers. Written informed consent was obtained from all the participants in the study according to the requirements of the ethical committee of each medical center.

The clinical and research activities being reported are consistent with the Principles of the Declaration of Istanbul as outlined in the 'Declaration of Istanbul on Organ Trafficking and Transplant Tourism'.

### Definitions and endpoints

CCr was estimated at baseline by Cockcroft-Gault equation: CCr = (140—age) * (weight in kilograms) * (0.85 if female) / (72 * serum creatintine (in mg dL^−1^)) [9]. Renal insufficiency was defined as CCr < 60 ml/min, and CCr < 30 ml/min was considered as severe renal insufficiency. PE severity was categorized according to the ESC/ERS guidelines to acute PE [6]. sPESI score was calculated for individuals accordingly [10]. A score point of 1 each was assigned for patients with any of the following conditions: age over 80 years, presence of cancer, chronic heart failure or chronic pulmonary disease, pulse rate ≥ 110 bpm, systolic blood pressure < 100 mmHg, and peripheral arterial oxygen saturation < 90% [10]. The total sPESI score was used to divide hemodynamically stable patients into intermediate-risk (sPESI ≥ 1) and low-risk patients (sPESI = 0). Considering the products of LMWH regimen (e.g., enoxaparin sodium, dalteparin sodium, nadroparin calcium) varied across different clinical centers, LMWH dosage was converted into IU/kg daily dose during data analysis and presented as adjusted dose (≤ 100 IU/kg/day) and conventional dose (> 100 IU/kg/day).

The primary endpoint of the study was in-hospital all-cause death. The secondary endpoints were (i) PE-related death, defined as death considered to be due to PE by autopsy or if the patients died shortly after objectively confirmed symptomatic PE and in the absence of alternative diagnosis [11]; (ii) Bleeding events, including major bleeding and clinically relevant non-major bleeding, were defined according to the criteria in the International Society of Thrombosis and Haemostatsis (ISTH) [12].

### Statistical analysis

Baseline patient characteristics were expressed in terms of descriptive statistics. Categorical variables were summarized as frequency (percentage). Continuous variables were presented as mean (standard deviation, SD) or median (interquartile range, IQR). *P* values were calculated by students’ t test, χ2 test or Fisher exact test among different renal function groups/LMWH dose group where appropriate. Logistic regression, adjusting for age and gender, was performed to explore the odds ratios for adverse outcomes, including death, PE related death, bleeding and major bleeding, in patients with 30 ml/min ≤ CCr < 60 ml/min and CCr < 30 ml/min, compared to those with CCr ≥ 60 ml/min, respectively. Univariable and multivariable regression among patients with renal insufficiency (CCr < 60 ml/min) were performed to explore the risk factors for in-hospital bleeding events. Factors including adjusted LMWH dose, cancer and length of hospital stay were included into multivariable regression to estimate the odds ratios (OR) and 95% confidence intervals (95% CIs), taking both clinical value and statistical significance into consideration. Kaplan–Meier curves were drawn to compare the cumulative rates of all-cause death and PE-related death in patients with different renal function groups respectively, and compared by log-rank test. All tests were two-sided and were considered statistically significant at a *p*-value of < 0.05. All analyses were performed using SAS 9.4 software (Cary, NC, USA).

## Results

### Baseline characteristics

**Fig. 1:**
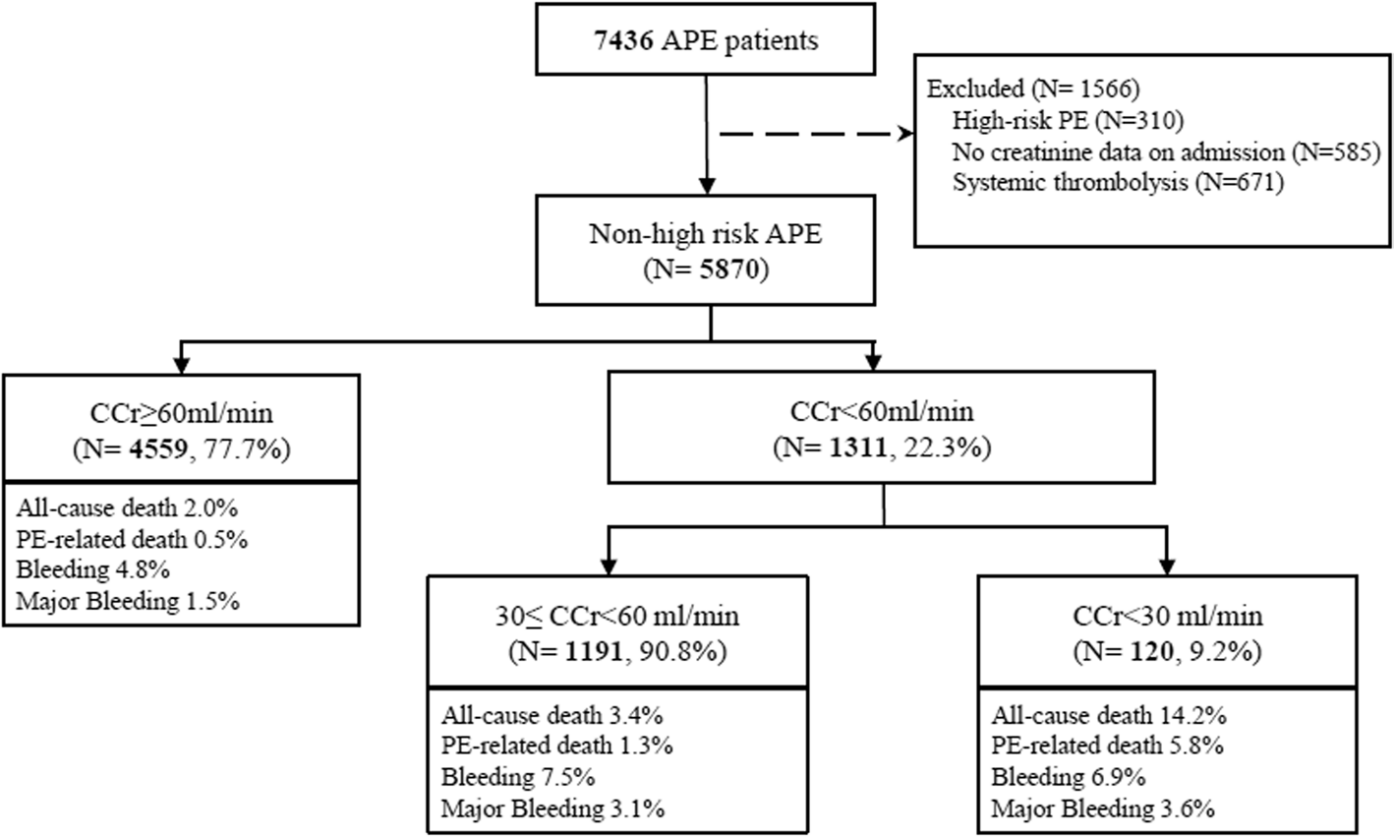
Flowchart. Abbreviations: APE, acute pulmonary embolism; CCr, creatinine clearance

Among the CURES cohort, a total of 5870 non-high-risk PE patients were enrolled into analysis. 1311 (22.3%) patients were identified to have renal insufficiency in admission. Among those patients, 1191 (90.8%) were 30 ≤ CCr < 60 ml/min and 120 (9.2%) were CCr < 30 ml/min (Fig. 1). Characteristics of patients with renal insufficiency are demonstrated in Table 1.

**Table 1:** Characteristics patients with acute PE by CCr level

Variable	≥ 60 ml/min *N* = 4559	30–60 ml/min *N* = 1191	< 30 ml/min *N* = 120	Total *N* = 5870	*P* value
**Demographic Features**					
Age, years	60.0 (49.3, 69.1)	75.5 (69.2, 80.3)	78.9 (70.6, 83.5)	63.6 (52.7, 73.4)	< .0001
Age > 65 (years)	1626 (35.7)	1003 (84.2)	104 (86.7)	2733 (46.6)	< .0001
Age > 80 (years)	168 (3.7)	309 (25.9)	49 (40.8)	526 (9.0)	< .0001
Female	2045 (44.9)	645 (54.2)	69 (57.5)	2759 (47.0)	< .0001
BMI, kg/m^2^	24.2 (22.2, 26.4)	22.4 (20.5, 24.8)	21.5 (19.5, 23.4)	23.8 (21.7, 26.0)	< .0001
**Comorbidities**					
Cardiovascular Disease	1759 (38.6)	736 (61.9)	80 (66.7)	2575 (43.9)	< .0001
Respiratory Diseases	938 (20.6)	388 (32.7)	36 (30.0)	1362 (23.2)	< .0001
Cancer	418 (9.2)	99 (8.4)	10 (8.3)	527 (9.0)	0.6466
Diabetes	444 (9.8)	167 (14.1)	24 (20.0)	635 (10.9)	< .0001
Neurological disease	425 (9.4)	182 (15.5)	27 (22.5)	634 (10.9)	< .0001
Chronic nephritis	20 (0.4)	20 (1.7)	11 (9.2)	51 (0.9)	< .0001
Nephrotic syndrome	40 (0.9)	14 (1.2)	5 (4.2)	59 (1.0)	0.0196
**Vital Signs & Laboratory Tests**					
Pulse ≥ 110 beats/min	345 (7.6)	97 (8.3)	14 (11.8)	456 (7.8)	0.2044
Respiratory Rate, times/min	20.0 (18.0, 22.0)	20.0 (18.0, 22.0)	20.0 (19.0, 23.0)	20.0 (18.0, 22.0)	0.0013
Systolic blood pressure, mmHg	125.0 (116.0, 140.0)	130.0 (118.0, 142.0)	133.0 (120.0, 147.5)	127.0 (117.0, 140.0)	< .0001
Elevated D-dimer	3574 (87.1)	951 (88.3)	97 (92.4)	4622 (87.4)	0.1668
Hemoglobin, g/L	131.0 (117.0, 143.0)	127.0 (115.0, 139.0)	115.0 (97.0, 129.0)	130.0 (116.0, 142.0)	< .0001
Platelet < 100 × 10^9^/L	225 (5.0)	88 (7.5)	14 (11.8)	327 (5.6)	< .0001
PaO_2_ < 60 mmHg	699 (17.8)	263 (25.2)	25 (24.8)	987 (19.4)	< .0001
Creatinine, μmol/L	66.7 (56.0, 78.5)	91.0 (77.1, 109.0)	159.5 (129.0, 212.6)	71.0 (59.0, 86.0)	< .0001
BUN, mmol/L	5.0 (3.9, 6.2)	6.6 (5.1, 8.4)	10.2 (7.6, 13.4)	5.2 (4.0, 6.9)	< .0001
BUN/Cr	18.4 (14.3, 23.3)	17.8 (14.1, 22.3)	15.7 (11.6, 19.9)	18.2 (14.2, 23.1)	< .0001
**Risk Stratification**					
sPESI ≥ 1	4501 (98.7)	1190 (99.9)	120 (100.0)	5811 (99.0)	< .0001
**Outcomes**					
All-cause death	91 (2.0)	40 (3.4)	17 (14.2)	148 (2.5)	< .0001
PE-related death	25 (0.5)	16 (1.3)	7 (5.8)	48 (0.8)	< .0001
Bleeding	155 (4.8)	65 (7.5)	6 (6.9)	226 (5.4)	0.0089
Major bleeding	47 (1.5)	26 (3.1)	3 (3.6)	76 (1.9)	0.0085
Length of hospital stay (days)	19.0 (12.0, 30.0)	18.0 (12.0, 30.0)	21.0 (13.0, 30.0)	19.0 (12.0, 30.0)	0.6522

Data were expressed as median (interquartile range) or number (proportion), where appropriate. *P* values were calculated by Kruskal–Wallis test, χ2 test or Fisher exact test

Abbreviations: *PE* pulmonary embolism, *CCr* creatinine clearance, *BMI* body mass index, *BUN* blood urea nitrogen, *Cr* creatinine, *SD* standard deviation, *sPESI* simplified pulmonary embolism severity index

### In-hospital outcomes of patients in different renal function groups

The rates of all-cause death, PE-related death, bleeding and major bleeding were all higher in patients with renal insufficiency than those without renal insufficiency (Fig. 1). After adjustment of age and gender, 30 ≤ CCr < 60 ml/min was significantly associated with PE-related death (OR 2.10, 95%CI 1.02–4.31), bleeding (OR 1.78, 95%CI 1.27–2.50) and major bleeding (OR 2.71, 95%CI 1.54–4.75); severe renal insufficiency was significantly associated with all-cause death (OR 5.11, 95%CI 2.80–9.31) and PE-related death (OR 9.34, 95%CI 3.59–24.25) (Supplementary Figure S1). The cumulative all-cause death rate and PE-related death rate among patients with severe renal insufficiency were also significantly higher than other groups (both log-rank *p* < 0.0001) (Supplementary Figure S2).

### Anticoagulant application in renal insufficient patients

**Fig. 2:**
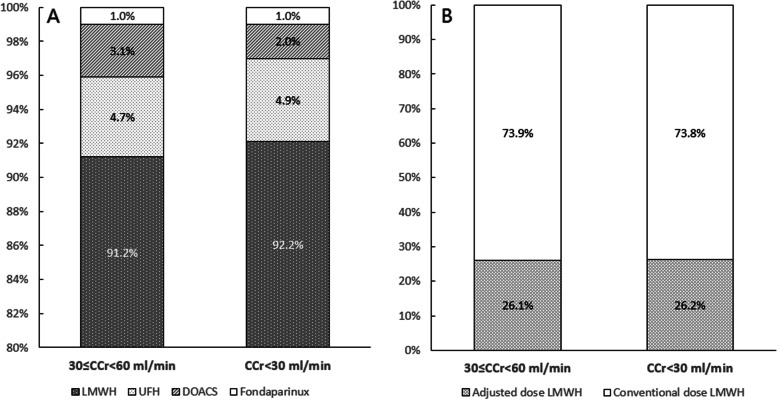
Anticoagulants application [Panel **A**] and LMWH dose distribution [Panel **B**] among PE patients with renal insufficiency. Abbreviations: CCr, creatinine clearance; DOACS, direct oral anticoagulants; PE, pulmonary embolism; LMWH, low molecular weight heparin; UFH, unfractured heparin

LMWH has been applied commonly in renal insufficient patients, even among patients with severe renal insufficiency (91.2% in 30 ≤ CCr < 60 ml/min and 92.2% in CCr < 30 ml/min). UFH was used in 4.7% and 4.9% patients with moderate and severe renal insufficiency, respectively. DOACs were used in 3.1% and 2.0% patients with moderate and severe renal insufficiency, respectively. Fondaparinux was applied in 1.0%, both in patients with moderate and severe renal insufficiency (Fig. 2A). Patients with complete records of LMWH dose (*N* = 1042) were further analyzed: of the patients who were initially anticoagulated with LMWH, 273 (26.2%) were admitted adjusted dose and 769 (73.8%) were admitted conventional dose. 26.1% in 30 ≤ CCr < 60 ml/min and 26.2% in CCr < 30 ml/min were prescribed adjusted dose of LMWH (Fig. 2B).

### Outcomes of renal insufficient patients with adjusted and conventional dose of LMWH

**Fig. 3:**
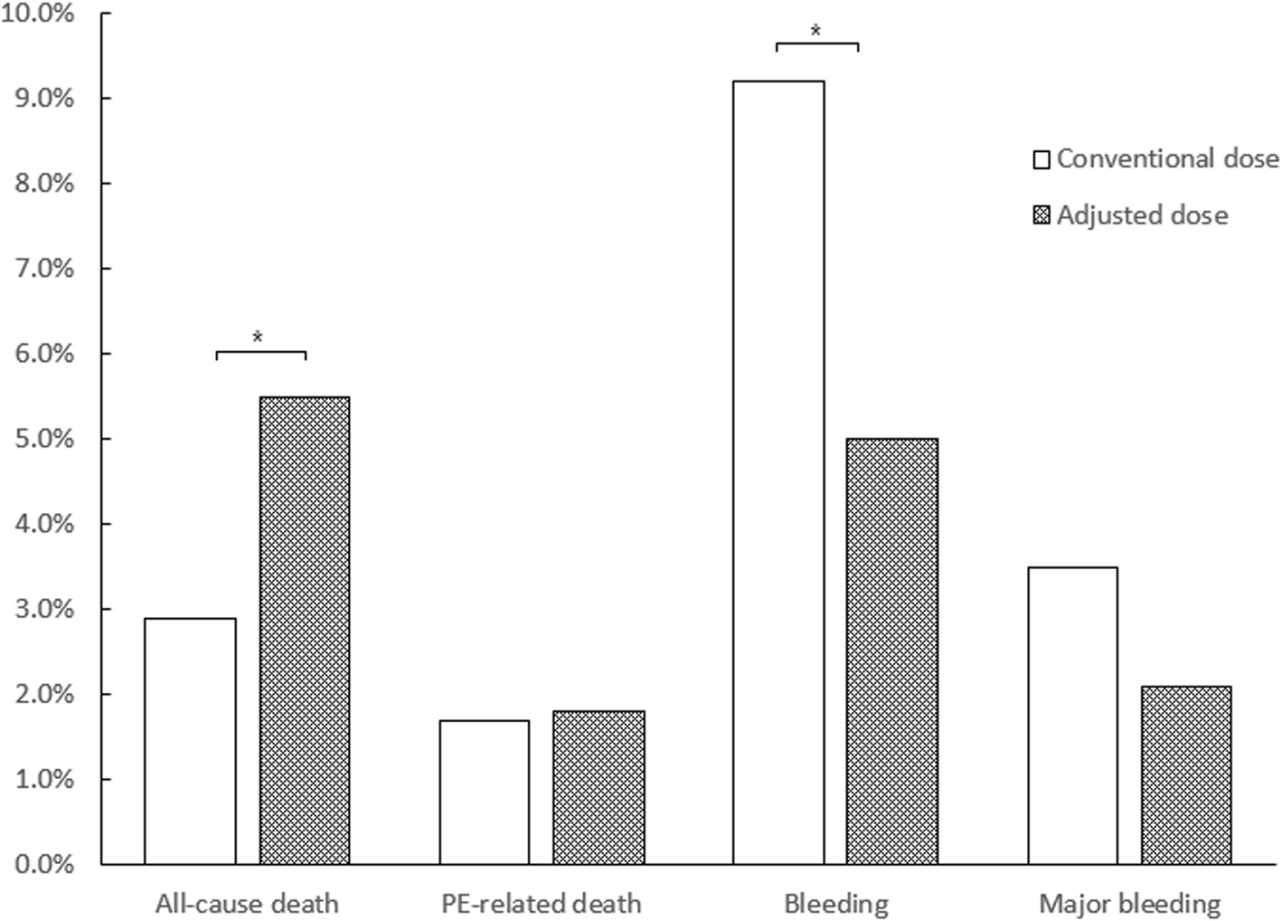
Rates of in-hospital outcomes of renal insufficient patients undertaken conventional and adjusted dose of LMWH. Note. LMWH, low molecular weight heparin. * *p* < 0.05

Baseline characteristics were compared between the two groups of patients: those with older age, history of respiratory diseases, and more slight symptoms of PE were more likely to be prescribed adjusted LMWH (Supplementary Table S1). Bleeding events during hospitalization occurred significantly more frequently among those who were undertaken conventional dose of LMWH (9.2% vs 5.0%, *p* = 0.0466), major-bleeding rate was also higher among those patients, but the difference was not statistically significant (3.5% vs 2.1%, *p* = 0.3082). The rate of in-hospital all-cause death was significantly higher in those with adjusted dose of LMWH than conventional dose (5.5% vs 2.9%, *p* = 0.0434), the rates of PE-related death were similar between those two groups of patients (1.8% vs 1.7%, *p* = 0.8787) (Fig. 3). The outcomes in more detailed groups of renal insufficiency were presented in Supplementary Table S2.

We further used multivariable logistic regression to identify the risk factors for in-hospital bleeding among patients with renal insufficiency. Adjusted dose of LMWH presented as protective factor for in-hospital bleeding (OR 0.62, 95%CI 0.27–1.00, *p* = 0.0496) and the risk of bleeding increased as length of hospital stay prolonged (OR 1.03, 95%CI 1.01–1.06, *p* = 0.0014 (Table 2).

**Table 2:** Univariable and Multivariable regression of risk factors for in-hospital bleeding events among patients with renal insufficiency (CCr < 60 ml/min)

	**Univariable**		**Multivariable**	
**Variable**	**OR and 95% Cl**	***P* value**	**OR and 95% Cl**	***P* value**
Adjusted LMWH dose	0.52 (0.27–1.00)	0.0500	0.52 (0.27–1.00)	0.0496
Age > 80 (years)	1.08 (0.63–1.85)	0.7801		
Female	1.04 (0.64–1.70)	0.8695		
BMI, kg/m^2^	0.94 (0.87–1.01)	0.0933		
Cancer	1.19 (0.53–2.69)	0.6762	1.36 (0.59–3.14)	0.4769
Pulse ≥ 110 bpm	0.62 (0.22–1.76)	0.3718		
Anaemia^a^	1.65 (1.00–2.75)	0.0523		
Platelet < 100 × 10^9^/L	1.21 (0.54–2.74)	0.6462		
Length of hospital stay (days)	1.03 (1.02–1.06)	0.0005	1.03 (1.01–1.06)	0.0014

OR and 95% CI were estimated by Logistic regression model

Abbreviations: *BMI* body mass index, *LMWH* low molecular weight heparin, *CCr* creatinine clearance, *OR* odds ratio, *95% CI* 95% confidence interval

^a^Anemia refers to hemoglobin < 120 g/L for male and < 110 g/L for female, respectively

## Discussion

In our study, more than one fifth non-high risk acute PE patients were found to have renal insufficiency in admission. Among renal insufficient patients, LMWH was commonly applied and mostly with unadjusted dose. Adjusted dose of LMWH was significantly associated with lower rate of in-hospital bleeding for renal insufficient patients. To our knowledge, this is the first study focusing on the dosage of LMWH in PE patients with renal insufficiency and the association with in-hospital outcomes at real-world setting.

In our study, renal function was presented as creatinine clearance calculated by Cockcroft-Gault formula, this was mainly because that creatinine clearance is often used to the indication of kidney function for adjustment of dosage requirements, as our purpose on analysis of LMWH dosage. Present studies have reported a prevalence of renal insufficiency/dysfunction around 27%-49% in patients with acute PE [13]. In our study, 22.3% of involved normotensive acute PE patients were identified as renal insufficiency, lower than previous studies. The difference in the prevalence may due to the different equations in the estimation of renal insufficiency/dysfunction or the population involved.

We revealed a significantly increased risk of bleeding in patients with renal insufficiency during hospitalization. The rates of bleeding and major bleeding in CCr < 30 ml/min group with conventional dose of LMWH were comparable with previous real-world studies and meta-analysis. RIETE study reported the rates of major bleeding: 6.4% in CCr < 30 ml/min, fatal bleeding 1.0% during the first 15 days [14] and 8.3% bleeding events were found in a recent meta-analysis focused on the use of LMWH in VTE patients with severe renal insufficiency [15].

The 2019 European Society of Cardiology/European Respiratory Society (ESC/ERS) guidelines of acute PE recommends UFH for patients with serious renal impairment (CCr ≤ 30 ml/min) and because that renal clearance is indirectly proportional to molecular weight, an adapted dosing scheme should be used while LMWH is prescribed in patients with CCr 15-30 ml/min [16]. Enoxaparin is the most commonly used LMWH and mostly studied, the 1-mg/kg QD regimen is recommended for severe CKD. There is no data for dalteparin and tinzaparin in severe CKD. For dosage adjustment purposes, it is recommended to monitor the activity of (anti-Xa level in order to avoid underdosage and achieve optimal therapeutic level, respectively. However, monitoring the activity of anti-Xa was not available in all healthcare providers, and dosing indications are results of either small-scale open-label studies, or analysis of CKD subgroups in randomized trials, adopted by guidelines, which, inevitably, are of low level of evidence [16]. In our study, a very high proportion of conventional dose LMWH was found in patients with renal insufficiency, including CCr < 30 ml/min.

Anticoagulation therapy among PE patients with renal insufficiency has been taken into consideration in recent real-world studies, The Global Anticoagulant Registry in the Field-Venous Thromboembolism (GARFILED-VTE) reported an up to 60% usage of parenteral therapy among VTE patients with moderate to severe CKD in the first month of treatment [17]. In RIETE study, the proportion of LMWH non-adherent management was as high as 20.8% for severe renal insufficiency, severe obesity and unstable PE patients and was related to high risk of death [7]. Another analysis of RIETE study found that most of the VTE patients with renal insufficiency received LMWH as initial therapy, with a mean daily dosage similar as the recommended dose for patients with normal renal function. Of note, the rates of major bleeding and fatal bleeding in patients with severe renal insufficiency were similar between those receiving UFH or LMWH, and no difference in mean LMWH doses was found between those patients who died and survived [18]. A newly developed risk score for predicting early major bleeding in acute PE had also identified renal dysfunction as one of the four core parameters [19]. Researchers inferred that dosage of anticoagulant might be a reason for patients with renal insufficiency to have higher risk of bleeding.

Limited evidence for anticoagulation for patients with renal insufficiency has been provided by RCTs, as severe renal impairment was among regular exclusion criteria for clinical trials. Renal Insufficiency Study (IRIS) compared full dose UFH and reduced dose of tinzaparin (175 IU/kg once daily) in renally impaired patients ≥ 70 years with acute DVT, the mortality favored UFH group and the rates of clinically relevant bleeding by day 90 were similar in the tinzaparin (11.9%) and UFH (11.9%) groups [20]. A post-hoc analysis of the CLOT study of cancer patients with renal impairment (CCr < 60 ml/min) showed that the bleeding rates were similar between dalteparin 200 IU/kg once daily and group and VKA [21]. A meta-analysis demonstrated that major bleeding increased when a standard therapeutic dose of enoxaparin was used (8.3% vs. 2.4%; odds ratio, 3.88 [CI, 1.78 to 8.45]) but may not increase when an empirically adjusted dose of enoxaparin is used (0.9% vs. 1.9%; OR 0.58 [CI, 0.09 to 3.78]) [15].

Our study innovatively analyzed the association between LMWH dosage and in-hospital outcomes for renal insufficient patients and a protective effect of adjusted dose LMWH for in-hospital bleeding events was demonstrated. The results emphasize the importance of LMWH dosage among renal insufficient patients, especially for safety regards. On the other hand, those who were administered adjusted dose of LMWH had significant higher rate of all-cause death during hospitalization. The reason would be that the complexity of background clinical status in renal insufficient patients leads to higher mortality or risk of fatal bleeding. This finding might alert physician to reduce the dosage of anticoagulant. Therefore, randomized clinical trials of larger sample of renal insufficient patients with longer follow-up time are required to investigate the relationship between treatment strategies and outcomes. We hypothesize that, for these patients, it is considerable to administer anticoagulants at a lower frequency or for a shorter time period. Dynamically monitor the risk of bleeding at follow-up period is also important.

Notably, our study found that the risk of bleeding increased as length of hospitalization prolonged. As the anticoagulation phase would last for at least 3 to 6 month, the prolonged hospitalization days means a longer observation time. Previous study reported an increasing risk of bleeding events after the first 15–30 days of anticoagulation in RIETE study, which was also proved by our study, indicating that the balance between efficacy and safety for those patients should be reassessed after acute phase of treatment [22].

There were a few limitations of our study: firstly, patients were not followed up during the study period and the long-term prognosis will be discussed in the following stages of CURES study, as described elsewhere [8]. Secondly, both products of LMWH regimen prescribed and dosage strengths in different hospitals varied (including enoxaparin sodium, dalteparin sodium, nadroparin calcium, etc.), thus the detail of particular regimen of LMWH was unavailable. The only way to make the result comparable was to re-estimate the LMWH dosage according to body weight in analysis. Thirdly, previous study reported evidence of recovery of renal injury during the spectrum of acute PE [23], which indicated a dynamic monitoring of creatinine is strongly required during follow-up period, to reassess prognosis (especially bleeding risk) and modify the dosage of drugs. In this study, our database only included the creatinine level at admission, so it was unavailable, so the recovery of renal function was unable to be observed. Fourthly, because DOACs were seldom administered during study period, the dosage and prognosis related to DOACs were not investigated in this study. However, even though DOACs are being widely prescribed as substitutes of traditional anticoagulants, LMWH still acts as the first-line drug for specific population, such as patients with cancer or pregnancy, according to ESC/ERS guidelines.

## Conclusions

The proportion of adjusted usage of LMWH was low and adjusted dose of LMWH was associated with lower risk of in-hospital bleeding for RI patients in real-world setting of PE treatment. Anticoagulation strategy for RI patients should be paid more attention and requires evidence of high quality.

## Supplementary Information

**Additional file 1: **Acknowledgments. **Supplementary Table S1.** characteristicsof renal insufficient patients undertaken adjusted and conventional dose ofLMWH. **Supplementary Table S2.** Outcomes of renalinsufficient non-high risk PE patients with adjusted and conventional dose ofLMWH. **Supplementary Figure S1. **Forest plot of odds ratios for death, PE related death, bleeding and majorbleeding by different renal function groups. **Supplementary Figure S2. **Kaplan-Meier curves of cumulative death rates [Panel A] and cumulative PErelated death rates [Panel B] by different renal function groups forin-hospital PE patients. 

## Data Availability

The datasets used and/or analyzed during the current study are available from the corresponding author on reasonable request.

## Abbreviations

AKI: Acute Kidney Injury
BMI: Body Mass Index
CCr: Creatinine Clearance
CI: Confidence Interval
CKD: Chronic Kidney Disease
CTPA: Computed Tomographic Pulmonary Angiography
CURES: China Pulmonary Thromboembolism Registry Study
DVT: Deep Vein Thrombosis
EDC: Electronic Data Capture
ESC/ERS: European Society of Cardiology/ European Respiratory Society
HR: Hazard Ratio
ICOPER: The International Cooperative Pulmonary Embolism Registry
ISTH: The International Society of Thrombosis and Haemostatsis
IU: International Unit
LMWH: Low Molecular Weight Heparin
N-GAL: Neutrophil Gelatinase-associated Lipocalin
NNH: Number Need to Harm
DOACs: Direct Oral Anticoagulants
PE: Pulmonary Embolism
RIETE: Registro Informatizado de Enfermedad TromboEmbólica
RV: Right Ventricle/Right Ventricular
SD: Standard Deviation
sPESI: Simplified Pulmonary Embolism Severity Index
UFH: Unfractionated Heparin
V/Q: Scan Ventilation-perfusion Lung Scintigraphy
VTE: Venous Thromboembolism
