# Supplementary material for: LMWHs dosage and outcomes in acute pulmonary embolism with renal insufficiency, an analysis from a large real-world study
Source: Thromb J. 2022 May 5;20:26. doi: 10.1186/s12959-022-00385-z (PMC9074331; doi:10.1186/s12959-022-00385-z)
Supplement: Supplementary file 1 — Additional file 1: Acknowledgments. Supplementary Table S1. characteristicsof renal insufficient patients undertaken adjusted and conventional dose ofLMWH. Supplementary Table S2. Outcomes of renalinsufficient non-high risk PE patients with adjusted and conventional dose ofLMWH. Supplementary Figure S1. Forest plot of odds ratios for death, PE related death, bleeding and majorbleeding by different renal function groups. Supplementary Figure S2. Kaplan-Meier curves of cumulative death rates [Panel A] and cumulative PErelated death rates [Panel B] by different renal function groups forin-hospital PE patients. [file 12959_2022_385_MOESM1_ESM.docx]

**Acknowledgments**

We appreciate the continuous support and contributions from the China Pulmonary Thromboembolism Registry Study(CURES) investigators**:** Zhonghe Zhang, Yingqun Ji, Jun An (The First Affiliated Hospital of Dalian Medical University), Yuanhua Yang, Jifeng Li (Beijing Chao-Yang Hospital, Capital Medical University), Qun Yi, Lan Wang, Haixia Zhou, Maoyun Wang (West China Hospital, West China School of Medicine, Sichuan University), Hong Chen, Xiaohui Wang (The First Affiliated Hospital of Chongqing Medical University), Baomin Fang, Xiaomao Xu, He Yang (Beijing Hospital), Zhihong Liu, Qin Luo (Fuwai Hospital, Chinese Academy of Medical Science; National Center for Cardiovascular Diseases), Mian Zeng, Xia Li (The First Affiliated Hospital, Sun Yat-Sen University), Ling Zhu, Yi Liu (Shandong Provincial Hospital), Kejing Ying, Guofeng Ma, Chao Yan (Sir Run Shaw Hospital, Zhejiang University School of Medicine), Zhenguo Zhai, Wanmu Xie, Jun Wan (China-Japan Friendship Hospital), Lixia Dong, Wei Zhou (Tianjin Medical University General Hospital), Chong Bai, Wei Zhang (Changhai Hospital), Liangxing Wang, Yupeng Xie, Xiaoying Huang (The First Affiliated Hospital of Wenzhou Medical University), Chen Qiu, Yazhen Li, Yingyun Fu, Shengguo Liu (Shenzhen People's Hospital), Shengqing Li, Jian Zhang, Xinpeng Han (Xijing Hospital), Qixia Xu, Xiaoqing Li, Yingying Pang, Beilei Gong (The First Affiliated Hospital of Bengbu Medical College), Ping Huang, Yanwei Chen, Jiming Chen (Shenzhen Sixth People's Hospital (Nanshan Hospital) Huazhong University of Science and Technology Union Shenzhen Hospital), Guochao Shi, Yongjie Ding (Ruijin Hospital Affiliated to Shanghai Jiaotong University School of Medicine), Zhaozhong Cheng, Li Tong (The Affiliated Hospital of Qingdao University), Zhuang Ma, Lei Liu (The General Hospital of Shenyang Military), Luning Jiang, Zhijun Liang (Affiliated Hospital of Jining Medical University), Chaosheng Deng, Minxia Yang, Dawen Wu (The First Affiliated Hospital of Fujian Medical University), Shudong Zhang, Lijun Kang (Yantaishan Hospital), Hong Chen, Fangfei Yu, Xuewei Chen (The Second Affiliated Hospital of Harbin Medical University), Dan Han, Shasha Shen (The First Affiliated Hospital of Kunming Medical University), Guohua Sun, Yutao Hou, Baoliang Liu (Zibo First Hospital), Xiaohong Fan, Wei Zhang (Nanjing Drum Tower Hospital), Ping Zhang, Ruhong Xu (Dongguan People's Hospital), Zaiyi Wang, Cunzi Yan (The First Affiliated Hospital of Xinjiang Medical University), Chunxiao Yu, Zhenfang Lu, Jing Hua (Beijing Jingmei Group General Hospital), Zhenyang Xu, Hongxia Zhang, Jinxiang Wang (Beijing Luhe Hospital, Capital Medical University), Xiaohong Yang, Ying Chen (People's Hospital of Xinjiang Uygur Autonomous Region), Yongjun Tang, Wei Yang (Xiangya Hospital Central South University), Nuofu Zhang, Linli Duan, Simin Qing, Chunli Liu (The First Affiliated Hospital of Guangzhou Medical University (Guangzhou Institute of Respiratory Health)), Juhong Shi, Junping Fan (Peking Union Medical College Hospital), Lian Jiang, Hongda Zhao, Chengying Liu (Jiangyin People's Hospital), Yadong Yuan, Xiaowei Gong (The Second Hospital of Hebei Medical University), Xinhong Zhang, Chunyang Zhang (The Sixth Medical Center of People's Liberation Army General Hospital), Shuyue Xia, Hui Jia, Yunxia Liu (Central Hospital Affiliated to Shenyang Medical College), Dongmei Zhang, Yuntian Ma (Tianjin Ninghe District Hospital), Lu Guo, Jing Zhang (Sichuan Academy of Medical Sciences & Sichuan Provincial People's Hospital), Lina Han, Xiaomin Bai (Handan First Hospital), Guoru Yang, Guohua Yu (Weifang Respiratory Disease Hospital), Ruian Yang, Jingyuan Fan (The First People's Hospital of Yunnan Province), Aizhen Zhang, Rui Jiang, Xueshuang Li, Yuzhi Wu, Jun Han (Shanxi Provincial People's Hospital), Jingping Yang, Xiyuan Xu, Baoying Bu (The Third Affiliated Hospital of Inner Mongolia Medical University), Chaobo Cui, Ning Wang (Harrison International Peace Hospital), Yipeng Ding, Heping Xu, Dingwei Sun (Hainan General Hospital), Yonghai Zhang, Jie Duo, Yajun Tuo (Qinghai Provincial People's Hospital), Xiangyan Zhang, Weijia Liu (Guizhou Provincial People's Hospital), Hongyang Wang, Yuan Wang, Aishuang Fu (North China University of Science and Technology Affiliated Hospital), Songping Huang, Qinghua Xu (Quanzhou First Hospital), Wenshu Chai, Jing Li (The First Affiliated Hospital of Jinzhou Medical University), Yanping Ye, Wei Hu, Jin Chen (Fu Xing Hospital, Capital Medical University), Bo Liu, Lijun Suo (Linzi District People's Hospital), Changcheng Guo, Ping Wang (Taiyuan Central Hospital), Jinming Liu, Qinhua Zhao (Shanghai Pulmonary Hospital), Qin Luo, Le Kang (The Third Affiliated Hospital of Xinjiang Medical University), Jianying Xu, Lifen Zhao, Mengyu Cheng, Wei Duan (Shanxi Academy of Medical Sciences, Shanxi Dayi Hospital), Qi Wu, Li (Tianjin Haihe Hospital), Ping Wang, Xiuqing He, Yueyue Li (The 306th Hospital of People's Liberation Army ), Gang Chen, Yunxia Zhao, Zixiao Liu (The Third Hospital of Hebei Medical University), Guoguang Xia, Tianshui Li, Nan Chen, Xiaoyang Liu (Beijing Jishuitan Hospital), Tao Bian, Yan Wu (Wuxi People's Hospital), Huiqin Yang, Xiaoli Tang (Xinjiang Uygur Autonomous Region Hospital of Traditional Chinese medicine), Yiwen Zhang (Anhui Chest Hospital), Faguang Jin, Ning Wang, Yanli Chen, Yanyan Li (Tangdu Hospital), Jing Li, Miaochan Lao (Guangdong Academy of Medical Sciences, Guangdong General Hospital), Shengqing Li, Liang Dong (Shanghai Huashan Hospital), Guangfa Zhu, Wenmei Zhang (Beijing Anzhen Hospital, Capital Medical University), Liangan Chen, Zhixin Liang (Chinese People's Liberation Army General Hospital (Medical School of Chinese People's Liberation Army), Liping Cui, Cenfeng Xia, Jin Zhang, Peng Zhang (General Hospital of Ningxia Medical University), Lianxiang Guo, Sha Niu, Sichong Yu (Jiaozuo Second People’s Hospital), Guangjie Liu, Xinmao Wang (Beijing Tongren Hospitall, Capital Medical University), Yanhua Lv, Zhenyu Liang, Shaoxi Cai, Shuang Yang (Nanfang Hospital), Xinyi Zhang, Jiulong Kuang (The Second Affiliated Hospital of Nanchang University), Yanyan Ding, Yongxiang Zhang (People's Hospital of Beijing Daxing District), Xuejun Guo, Yanmin Wang (Xinhua Hospital Affiliated to Shanghai Jiaotong University School of Medicine), Jialie Wang, Ruimin Hu (Inner Mongolia People's Hospital), Lin Ma (The First Affiliated Hospital of Nanchang University), Yuan Gao, Rui Zheng (Shengjing Hospital of China Medical University), Zhihong Shi, Hong Li (The First Affiliated Hospital of Xi'an Jiaotong University), Yingqi Zhang, Guanli Su (The First Hospital of Hebei Medical University), Zhiqiang Qin, Guirong Chen (The People's Hospital of Guangxi Zhuang Autonomous Region), Xisheng Chen, Zhiwei Niu (The Hospital of Shunyi District Beijing), Jinjun Jiang, Shujing Chen (Zhongshan Hospital, Fudan University), Tiantuo Zhang, Hongtao Li, Jiaxin Zhu, Yuqi Zhou (The Third Affiliated Hospital, Sun Yat-Sen University), Yinlou Yang, Jiangtao Cheng (Yue Bei People's Hospital), Jie Sun, Yanwen Jiang (Beijing Shijitan Hospitall, Capital Medical University), Jianhua Liu, Yujun Wang (Beijing Huairou Hospital of University of Chinese Academy of Sciences), Ju Yin, Lanqin Chen (Beijing Children's Hospital, Capital Medical University), Min Yang, Ping Jiang, Hongbo Liu (Tianjin First Central Hospital), Guohua Zhen, Kan Zhang (Tongji Hospital, Tongji Medical College of Huazhong University of Science and Technology), Yixin Wan, and Hongyan Tao (Lanzhou University Second Hospital).

**Supplementary Table S1 characteristics of renal insufficient patients undertaken adjusted and conventional dose of LMWH**

| **Variable** | **Adjusted dose N = 273** | **Conventional LMWH N = 769** | **P** |
| --- | --- | --- | --- |
| Age > 80 (years) | 85 (31.1) | 182 (23.7) | 0.0152 |
| Female | 135 (49.5) | 419 (54.5) | 0.1520 |
| BMI, kg/m^2^ | 22.8 (20.8, 25.2) | 22.2 (20.3, 24.7) | 0.0644 |
| Cardiovascular Disease | 173 (63.4) | 476 (62.1) | 0.7012 |
| Respiratory Diseases | 103 (37.9) | 236 (30.8) | 0.0319 |
| Cancer | 22 (8.1) | 68 (8.9) | 0.6829 |
| Diabetes | 43 (15.9) | 106 (13.8) | 0.4131 |
| Neurological disease | 40 (14.8) | 116 (15.2) | 0.8550 |
| Cough | 122 (44.7) | 393 (51.2) | 0.0630 |
| Expectoration | 105 (38.5) | 307 (40.0) | 0.6499 |
| Fever | 37 (13.6) | 105 (13.7) | 0.9550 |
| Dyspnea | 181 (66.3) | 590 (76.9) | 0.0006 |
| Precordial Pain | 66 (24.2) | 176 (22.9) | 0.6798 |
| Pleural Pain | 20 (7.3) | 95 (12.4) | 0.0221 |
| Hemoptysis | 14 (5.1) | 63 (8.2) | 0.0945 |
| Palpitation | 20 (7.3) | 124 (16.2) | 0.0003 |
| Syncope | 23 (8.4) | 84 (11.0) | 0.2379 |
| Pulse ≥ 110, beats/min | 22 (8.1) | 67 (8.8) | 0.7054 |
| Respiratory Rate | 20.0 (18.0, 22.0) | 20.0 (19.0, 22.0) | 0.1521 |
| Systolic blood pressure, mmHg | 130.0 (119.0, 145.0) | 130.0 (118.0, 141.0) | 0.8588 |
| Elevated D-dimer | 228 (88.7) | 618 (89.2) | 0.8396 |
| Anaemia | 74 (27.6) | 193 (25.5) | 0.4905 |
| Platelet < 100×10^12^/L | 19 (7.1) | 51 (6.7) | 0.8435 |
| PaO_2_ < 60 mmHg | 59 (25.4) | 180 (25.9) | 0.8789 |

Notes. Data were expressed as mean (SD), median (interquartile range) or number (proportion), where appropriate. P values were calculated by student’s t test, Kruskal-Wallis test, χ2 test or Fisher exact test.

Abbreviations: PE, pulmonary embolism; CCr, creatinine clearance; BMI, body mass index; BUN, blood urea nitrogen; Cr, creatinine; SD, standard deviation; sPESI, simplified pulmonary embolism severity index.

**Supplementary Table S2. Outcomes of renal insufficient non-high risk PE patients with adjusted and conventional dose of LMWH**

| **CCr Group** | **LMWH dose** | **All-cause death** | **PE-related death** | **Bleeding** | **Major bleeding** |
| --- | --- | --- | --- | --- | --- |
| CCr ≥60 ml/min | Conventional dose | 37 (1.4%) | 8 (0.3%) | 94 (5.5%) | 25 (1.5%) |
|  | Adjusted dose | 25 (2.2%) | 7 (0.6%) | 45 (4.7%) | 13 (1.4%) |
|  | p value | 0.1269 | 0.2604 | 0.3655 | 0.8660 |
| 30 ≤ CCr < 60 ml/min | Conventional dose | 15 (2.1%) | 8 (1.1%) | 43 (9.1%) | 16 (3.5%) |
|  | Adjusted dose | 11 (4.4%) | 4 (1.6%) | 12 (5.4%) | 5 (2.3%) |
|  | p value | 0.0701 | 0.5242 | 0.1303 | 0.4860 |
| CCr < 30 ml/min | Conventional dose | 7 (10.8%) | 5 (7.7%) | 5 (10.9%) | 2 (4.4%) |
|  | Adjusted dose | 4 (17.4%) | 1 (4.4%) | 0 (0.0%) | 0 (0.0%) |
|  | p value | 0.468 | 1.000 | 0.3101 | 1.000 |


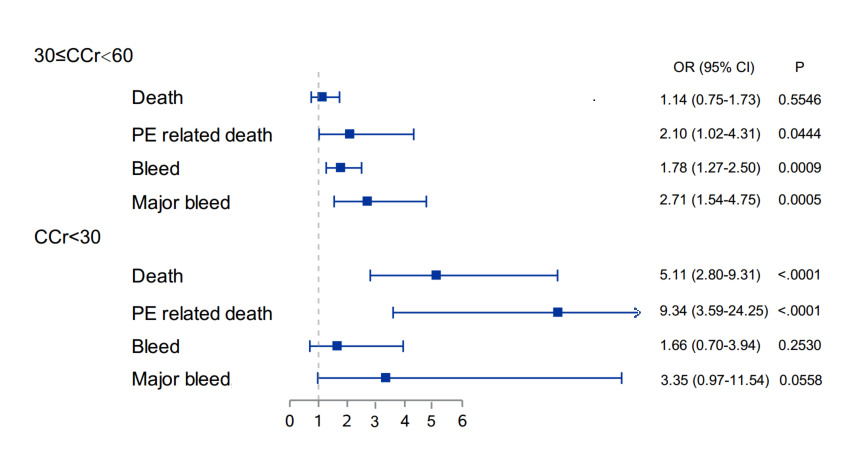


**Supplementary Figure S1. Forest plot of odds ratios for death, PE related death, bleeding and major bleeding by different renal function groups.**

Note. Odds ratios were estimated by Logistic regression, adjusting for age and gender, taking patients with CCr ≥ 60 ml/min as reference.

Abbreviations: CCr, creatinine clearance; OR, odds ratio; PE, pulmonary embolism.


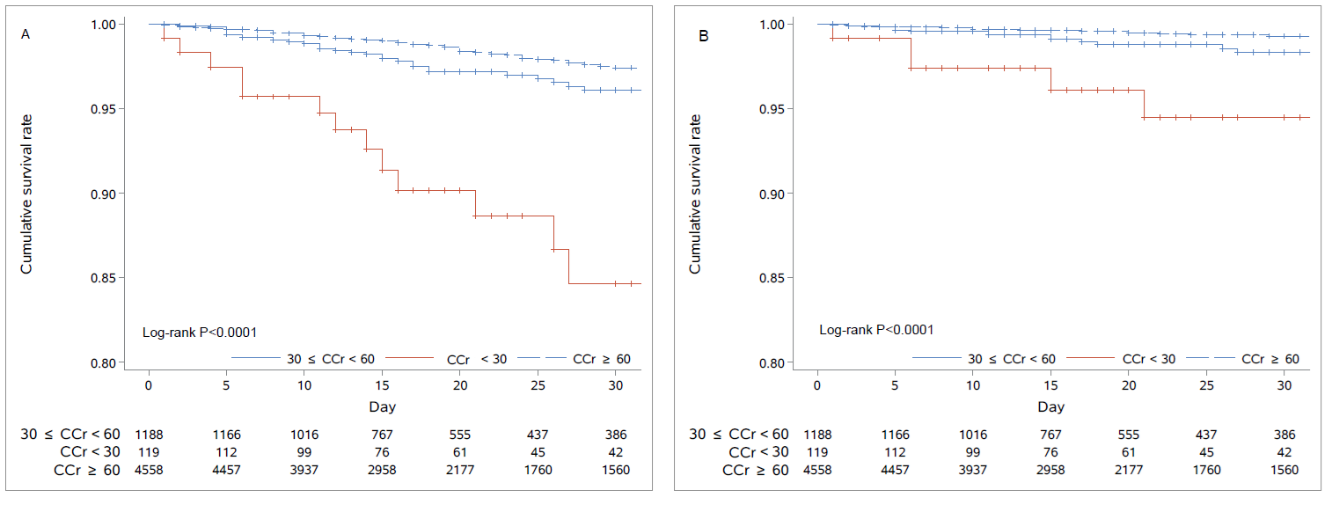


**Supplementary Figure S2. Kaplan-Meier curves of cumulative death rates [Panel A] and cumulative PE related death rates [Panel B] by different renal function groups for in-hospital PE patients.**

Note. P values were estimated by log-rank tests.

Abbreviations: CCr, creatinine clearance; PE, pulmonary embolism.
